# Combining TNFR2-Expressing Tregs and IL-6 as Superior Diagnostic Biomarkers for High-Grade Serous Ovarian Cancer Masses

**DOI:** 10.3390/cancers15030667

**Published:** 2023-01-21

**Authors:** Nirmala Chandralega Kampan, Apriliana Ellya Ratna Kartikasari, Cyril Deceneux, Mutsa Tatenda Madondo, Orla M. McNally, Katie Louise Flanagan, Norhaslinda A. Aziz, Andrew N. Stephens, John Reynolds, Michael A. Quinn, Magdalena Plebanski

**Affiliations:** 1 Department of Immunology & Pathology, Faculty of Medicine, Nursing and Health Sciences, Monash University, Level 6, The Alfred, Commercial Road, Melbourne, VIC 3181, Australia; 2 Oncology Unit, Royal Women’s Hospital, 20 Flemington Road, Parkville, VIC 3052, Australia; 3 Department of Obstetrics and Gynaecology, Faculty of Medicine, Universiti Kebangsaan Malaysia, Kuala Lumpur 56000, Malaysia; 4 School of Health and Biomedical Sciences, RMIT University, Melbourne, VIC 3083, Australia; 5 Department of Obstetrics and Gynaecology, Melbourne University, Parkville, VIC 3052, Australia; 6 Tasmanian Vaccine Trial Centre, Clifford Craig Foundation, Launceston General Hospital, Launceston, TAS 7250, Australia; 7 School of Health Sciences and School of Medicine, University of Tasmania, Hobart, TAS 7005, Australia; 8 Centre for Cancer Research, Hudson Institute of Medical Research, Clayton, VIC 3168, Australia; 9 Department of Molecular and Translational Sciences, Monash University, Clayton, VIC 3800, Australia; 10 Epworth Research Institute, Epworth Healthcare, Richmond, VIC 3121, Australia; 11 Biostatistics Consulting Platform, Faculty of Medicine, Nursing and Health Sciences, Monash University, Level 6, The Alfred, Commercial Road, Melbourne, VIC 3181, Australia

**Keywords:** progression-free survival, interleukin 6, high-grade serous ovarian cancer, epithelial ovarian cancer, tumour necrosis factor 2 receptor, regulatory T cells, inflammatory soluble biomarkers, platinum resistance

## Abstract

**Simple Summary:**

High-grade serous ovarian cancer (HGSOC) remains a lethal malignancy. There is an urgent need to establish whether a mass is benign or malignant prior to surgery. The HGSOC microenvironment harbours a mixture of immunosuppressive and inflammatory immune parameters that correlate independently with disease progression; however, their relationship is not well understood. We hypothesised that the inclusion of such diverse biomarkers would improve the prediction of ovarian malignancy. We quantified 29 soluble factors in blood, as well as the proportions of circulating T cell subsets using 16 phenotypic markers by multiparameter flow cytometry, in patients with suspected ovarian cancer or volunteers undergoing ovarian cancer risk reduction surgery. Out of all the soluble and cellular subsets tested, a combination of tumour necrosis factor receptor type 2 (TNFR2)-expressing regulatory T cells (Tregs) and interleukin 6 (IL-6) showed superior predictive ability over the biomarkers currently used to discriminate between benign and malignant tumors. We propose combining soluble and cellular circulating biomarkers as a useful approach to improve cancer diagnoses.

**Abstract:**

We hypothesised that the inclusion of immunosuppressive and inflammatory biomarkers in HGSOC patients would improve the sensitivity and specificity of the preoperative marker prediction of malignancy in patients with ovarian masses. We tested a panel of 29 soluble immune factors by multiplex bead immunoassay and 16 phenotypic T cell markers by flow cytometry in pre-treatment blood samples from 66 patients undergoing surgery for suspected ovarian cancer or ovarian cancer risk reduction. The potential diagnostic utility of all parameters was explored using Volcano plots, principal component analysis (PCA) and receiver operator characteristic (ROC) analysis. We also assessed the effect of culturing PBMCs from 20 healthy donors in the presence of malignant ascites fluid. The combination of TNFR2^+^ Tregs and IL-6 in the pre-treatment blood of patients with advanced HGSOC effectively discriminated patients with benign or malignant ovarian masses. *In vitro* culturing of the PBMCs of healthy donors in malignant ascites promoted an increase in TNFR2-expressing Tregs, which were decreased following blockade with IL-6 or STAT3 activity. Pre-treatment serum IL-6 and peripheral blood TNFR2^+^ Tregs may be potential clinical biomarkers that can discriminate patients with malignant compared to benign ovarian cancer masses, and the relationship between IL-6 and TNFR2^+^ Treg is likely to be mediated via the STAT3 signalling pathway.

## 1. Introduction

High-grade serous ovarian cancer (HGSOC) is the most common epithelian subtype of ovarian cancer and accounts for up to 70–80% of ovarian cancer deaths due to its aggressive nature and advanced presentation [1,2,3]. In Australia, advances in treatment and better multidisciplinary care have led to an improvement in the 5-year survival rate from 33% in 1982–1987 to 48% in 2000–2006 [4], still significantly lower than breast cancer survival, in part due to the earlier diagnosis of the latter by organised screening [4].

A definitive predictive biomarker for the early detection of ovarian cancer in asymptomatic women has not yet been identified. The accurate pre-operative assessment of ovarian masses allows timely referral to specialised centres for optimal surgical treatment if malignancy is suspected, leading to improved survival [5].

Standard investigation tools currently in place to delineate benign from malignant tumors include clinical examination, imaging, and assays of tumour markers including CA125 and HE4. Serum CA125, a glycoprotein antigen found elevated in various cancers [6,7], has low sensitivity and low specificity when used alone, is not expressed in 20% of ovarian cancers [8,9,10], and can be elevated in many medical diseases and non-malignant conditions [11]. Additional diagnostic methods, such as the examination of masses with ultrasound, may improve diagnostic accuracy but lack sensitivity and specificity. Several grading methods have been developed in an attempt to improve the preoperative discriminating between malignant and benign pelvic tumours [12]. Some solely consider the ultrasound approach, while others also include menopausal status, serum marker level, and/or colour Doppler in addition to ultrasound examination [12]. Human epididymis protein 4 [13], a transcript of the WFDC2 gene on chromosome 20, is a relatively new serum biomarker for the detection of ovarian cancer [14]. HE4, originally found to be expressed in human epididymis, can also be expressed in normal tissues including the respiratory and reproductive tracts [15]. HE4 was reported as a promising serum biomarker following its detection in ovarian cancer tissue, mainly serous [16,17], achieving higher sensitivity than CA125 alone, with a sensitivity of 72.9% and specificity of 95% [18,19,20]. The ROMA algorithm, which incorporates HE4 concentration with CA125 levels and menopausal status, has been shown to successfully triage 93.8% of ovarian cancer patients correctly as high risk [21]. The disadvantage of this algorithm is the use of HE4, which is expensive and not readily available in developing or undeveloped facilities. None of these tools are very sensitive or specific for detecting malignancy when considered separately.

The recent genetic mapping of high-grade serous ovarian cancer by the Cancer Genome Atlas Network [22] into four subtypes based on mRNA and miRNA expression and DNA methylation into immunoreactive, differentiated, proliferative and mesenchymal types has strongly linked ovarian cancer to parameters of the immune system [22]. Ovarian cancer masses, an immunogenic type of tumour, are infiltrated with immune cells and cytokines, particularly immunosuppressive cells such as regulatory T cells (Tregs) as well as inflammatory soluble factors [23,24]. This unique milieu contributes to immune escape by helping tumour cells evade host immunosurveillance so that they can continue growing without restriction [23,25,26]. In ovarian cancer, similar to other cancers, the immune system is hampered in controlling the tumour due to the presence of Tregs that inhibit effector T cell (Teff) mediated anti-tumour responses [25]. Cytokines (IL-6, TNF-α, IL-10, TGF-*β*) and chemokines (CCL2, CCL4, CXCL10) involved in the ovarian cancer microenvironment have also been shown to contribute to the development of carcinogenesis [27,28,29,30,31].

Ovarian cancer cells have constitutively activated signals including signal transducer and activator of transcription 3 (STAT3) [32], and nuclear factor kappa-light-chain-enhancer of activated B cells (NFκB) signalling, the elevation of which contributes to cancer cell resistance to chemotherapeutic agent-induced apoptosis [33]. Numerous studies analysing molecular mechanisms of platinum resistance in ovarian cancer have reported that the over-expression of IL-6 is one of the contributing factors for chemo-resistance and poor prognosis via STAT3-mediated activation [34,35]. The neutralisation of IL-6 and/or disruption of the STAT3 pathway may enhance the therapeutic efficacy of platinum-based chemotherapy and improve survival [30,36,37,38]. IL-6 is a pleiotropic cytokine, an essential biomarker in the cytokine cascade that is involved in the initiation and regulation of inflammation [39]. Elevated IL-6 in ascites and in the serum of patients with advanced ovarian cancer has been most strongly correlated with poor survival [40,41], as it has in multiple other cancers [29].

Tumour necrosis factor receptor 2 (TNFR2) belongs to the death receptors family and plays an important role in regulating apoptosis, cellular growth and proliferation via activation of the NFκB pathway. The expression of TNFR2 on the surface of Tregs is reported to identify the maximally suppressive and functional Treg population in both mice and humans [42,43]. Accumulated Tregs, particularly TNFR2-expressing Tregs in ascites and tissue, are higher in patients with ovarian cancer, and are linked to advanced disease and poor prognosis [25,44].

We hypothesised that the inclusion of inflammatory and immunosuppressive immune markers, both soluble and cell-mediated, found in the ovarian cancer tumour microenvironment may allow the identification of new biomarker(s) to help improve the detection of HGSOC.

## 2. Materials and Methods

### 2.1. Trial Design and Patient Details

This cross-sectional study was approved by the Human Research Ethics Committee (HREC) of the Royal Women’s Hospital, Melbourne: Immunity and Ovarian Cancer trial (Project 13/32). All procedures complied with the tenets outlined in the Declaration of Helsinki and the Good Clinical Practice Guidelines. A total of 80 women undergoing ovarian removal surgery and who fulfilled the study inclusion criteria were initially recruited following informed written consent. Informed consent was obtained from all subjects involved in the study.

**Figure 1:**
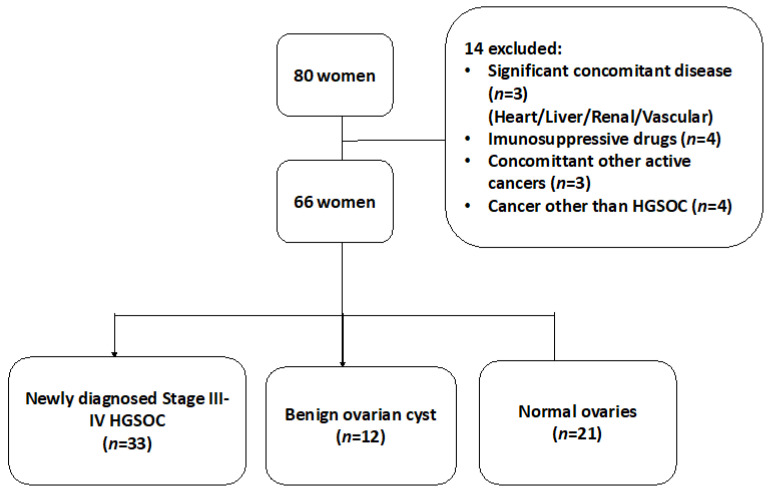
A flowchart of our cross-sectional study design for patients with suspected ovarian mass.

Fourteen women were excluded from the study and the reasons for exclusion are shown in Table 1. Sixty-six women were finally enrolled and constituted the final study population. In total, 33 women had newly diagnosed HGSOC, 19 women had benign ovarian masses and the control group consisted of 21 women undergoing risk reduction surgery for a known genetic mutation, e.g., breast cancer gene [45], Lynch syndrome or a strong family history of ovarian and/or breast cancer (Figure 1).

Comprehensive clinical information, including age, self-reported menopausal status, pre-existing conditions or medications, and any prior history of malignancy were obtained from de-identified patient medical records. Venous blood samples were obtained from patients prior to any surgical or chemotherapy treatment. Ascites samples were also collected either during peritoneal tapping prior to chemotherapy or at the time of surgery. Baseline blood components, serum CA125 levels and pelvic ultrasound reports were collected for all patients. Following surgery, relevant documentation on final diagnosis, surgical staging findings and a thorough histological assessment of tumour type, stage and grade following multidisciplinary team consensus were prospectively collected. Patients’ cancers were staged according to the criteria of the International Federation of Gynaecology and Obstetrics [46]. Patient details immediately relevant to this study are provided in Table 2. For in vitro cultures, 20 buffy coats were obtained from blood donated by healthy adult females acquired at the Australian Red Cross Blood Bank Service.

### 2.2. Serum Isolation

Whole blood of patients was collected in serum separation tubes. The serum was isolated within 3 h of blood collection by centrifugation of the tubes at 1000× *g* for 10 min. Following the removal of cellular and protein debris, the serum was aliquoted and stored at −80 °C until later use.

### 2.3. Ascites Supernatant Isolation

Ascites samples from ovarian cancer patients were first filtered through a 100 μm cell strainer and centrifuged to remove the cellular component. The cell-free supernatant layer of the ascites was collected and stored at −80 °C until use.

### 2.4. Isolation of Peripheral Blood Mononuclear Cells

Peripheral blood mononuclear cells (PBMCs) were isolated within 24 h of blood collection using the Ficoll (Amersham Pharmacia Biotech, Uppsala, Sweden) density gradient centrifugation method. The isolated PBMCs were then suspended in a freeze medium containing 10% DMSO (Sigma-Aldrich, St. Louis, MI, USA) and 90% heat-inactivated foetal calf serum (GIBCO, Life Technologies, USA) or 90% human AB serum (Sera Laboratories International, Sussex, UK) and frozen at a speed of 1 °C/min to −80 °C and subsequently stored in liquid nitrogen. Upon thawing, each vial of the frozen PBMCs was thawed rapidly in a 37 °C water bath and re-suspended gently in AIM-V media (Life Technologies, Grand Island, NY, USA) supplemented with 5% normal human serum (HS, Sigma-Aldrich, St. Louis, MI, USA) (complete AIM-V media).

### 2.5. Multiplex Bead Immunoassays

Multiplex magnetic bead immunoassay kits were used to simultaneously measure the concentrations of 28 analytes in a single sample. The concentrations of cytokines (GM-CSF, IFN-α, IFN-γ, IL-1β, IL-1RA, IL-2, IL-2R, IL-4, IL-5, IL-6, IL-7, IL-8, IL-10, IL-12 (p40/p70), IL-13, IL-15, IL-17 and TNF-α) and chemokines (RANTES, Eotaxin, CCL2/MCP-1, CCL3/MIP-1alpha, CCL4/ MIP-1beta, CXCL9/MIG, CXCL10/IP-10) were quantified using human 25-plex panel (Invitrogen™, Thermo Fisher Scientific, Vienna, Austria). This 25-plex panel was further combined with single-plex cytokine beads as per manufacturer’s protocol to measure the sTNFRII and CCL22/MDC concentrations. The concentration of HE4 was quantified using a separate human pre-mixed magnetic Luminex assay plate (R&D). The concentration of TGF-β was measured separately using a single-plex bead kit (Invitrogen™, Thermo Fisher Scientific, Vienna, Austria) as the serum samples required acidification to remove the latency-associated peptide from TGF-β1 prior to use in the assay.

Prior to analysis, all serum samples were completely thawed, clarified by centrifugation (1000× *g* for 10 min) and filtered. The serum samples were randomly assigned and were analysed in duplicates to avoid assay bias and to determine inter-assay differences. A standard curve was made by serially diluting the human cytokine standard cocktail in assay diluent. A total of 100 μL of each standard, 50 μL of patient sera mixed with 50 μL of assay diluent, was added to 96-well filter-bottom microplates together with 50 μL of incubation buffer and 25 μL of antibody-coated beads. The plates were sealed and incubated for 2 h on an orbital shaker at 500–600 rpm in the dark at room temperature. Wells were washed twice using a hand-held magnetic separator. A cocktail of biotinylated secondary antibodies was added, and the microplates were incubated for an hour in the dark on a microtiter shaker. After this step, washing was performed twice with a hand-held magnetic separator, followed by the addition of streptavidin–phytoerythrin and further incubation under agitation for 30 min in the dark at room temperature. The plates were then washed three times and the microspheres contained within each well were resuspended with 100 μL of wash solution. The plates were read and analysed using Luminex^®^ 200TM analyser (Luminex Corp, Austin, TX, USA) as per standard protocol. For each analyte, 100 beads were analysed, and the median fluorescence intensity was determined. Analysis of median fluorescence intensities was performed using five-parameter logistic curve fitting to the standard concentration of the analyte. The inter-assay variability of each assay was 2% to 8% and the intra-assay variability was 2% to 7%.

### 2.6. In-Vitro Conditioning with Ascites

PBMCs from healthy donors were cultured in a 96-well culture plate at a final concentration of 2 × 10^6^ cells/mL with 150 μL/well of either complete AIM V medium alone or with 50% ascites supernatant (obtained from patient ascites via centrifugation). The cells were then incubated in a humidified incubator at 37 °C with 5% CO_2_. After 48 h, the cells were harvested, antibody-labelled and further analysed by flow cytometry.

### 2.7. In Vitro Blockade of Cytokines within Ascites with Monoclonal Antibodies

Peripheral blood mononuclear cells from healthy donors were isolated by Ficoll density centrifugation and incubated in vitro in either complete AIM V media or cell-free ascites from an advanced EOC patient. STAT3 activity was inhibited by the addition of 50 μM S3I-201 (Santa Cruz Biotechnology, Heidelberg, Germany) into the culture at day 0. In untreated media and ascites, vehicle control DMSO at 0.05% was added at day 0. PBMCs from healthy donors were then added into a 96-well culture plate with 150 μL/well of either complete AIM V media or with 50% ascites supernatant (treated or untreated) at a final concentration of 2 × 10^6^ cells/mL. Following 48 h of incubation in a humidified incubator at 37 °C with 5% CO2, cells were washed and labelled for surface markers using the following antibodies: Anti-CD3 BV650 (BD Pharmingen, San Diego, CA, USA) and anti-CD8 FITC (BD Pharmingen, San Diego, CA, USA); anti-CD4 APC-Cy7 (BD Pharmingen, San Diego, CA, USA), anti-CD25 PECF584 (BD Pharmingen, San Diego, CA, USA) and anti-TNFR2 Biotin-Streptavidin AF700 (BD Pharmingen, San Diego, CA, USA). Following fixation and permeabilisation, intracellular staining was performed to detect FOXP3 and phosphorylated STAT3 using anti-FOXP3 APC (eBioscience, San Diego, CA, USA) and anti-STAT3 Phospho (Tyr705) BV421 (Biolegend, San Diego, CA, USA), respectively, and the cells were then analysed by flow cytometry.

### 2.8. Flow Cytometric Analysis

We evaluated a total of 16 (surface and intracellular) phenotypic markers by multicolour flow cytometry, mainly expressed by key regulatory T cell (Tregs) and effector T cell (Teffs) phenotypes. These phenotypic markers were selected based on the literature citing the abundance of these markers in the ovarian cancer tumour microenvironment, and/or their association with drug resistance and poor prognosis [22,25,29,31,33,44,47,48,49,50,51,52,53]. These phenotypes constituted six different groups of immune markers categorised according to their function or activity: immunosuppressive (TNFR2, PD-L1, CTLA-4, GARP), activation (Ki67, CD69, CD38), apoptosis (CD95), exhaustion (PD1), STAT3 related (IL-6R and STAT3) and migratory-related marker (CCR4, CCR7, CXCR3, CCR6, CCR2).

To determine the frequency and phenotype of T cell populations, up to 1 million cells from the PBMC of patients were stained with the following fluorescently labelled antibodies, split into three panels: Panel A: Anti-CD3 BV650 (BD Pharmingen, San Diego, CA, USA) and anti-CD8 FITC (BD Pharmingen, San Diego, CA, USA); anti-CD4 APC-Cy7 (BD Pharmingen, San Diego, CA, USA), and anti-CD25 PECF584 (BD Pharmingen, San Diego, CA, USA); anti-TNFR2 Biotin-Streptavidin AF700 (BD Pharmingen, San Diego, CA, USA), anti-IL-6R PE-Cy7 (Biolegend, San Diego, CA, USA), CD127 PerCP-Cy5.5 (Biolegend, San Diego, CA, USA), anti-PD1 PE (BD Pharmingen, San Diego, CA, USA), anti-PDL1 BV711 (Biolegend, San Diego, CA, USA), anti-CD95 BV786 (BD Pharmingen, San Diego, CA, USA), anti-CTLA-4 BV605 (Biolegend, San Diego, CA, USA). Panel B: Anti-CD3 BV650 (BD Pharmingen, San Diego, CA, USA) and anti-CD8 FITC (BD Pharmingen, San Diego, CA, USA); anti-CD4 APC-Cy7 (BD Pharmingen, San Diego, CA, USA), anti-CD25 PECF584 (BD Pharmingen, San Diego, CA, USA), anti-TNFR2 Biotin-Streptavidin AF700 (BD Pharmingen, San Diego, CA, USA), anti-IL-6R PE-Cy7 (Biolegend, San Diego, CA, USA), anti-CCR6 BV605 (Biolegend, San Diego, CA, USA), anti-CCR7 BV786 (BD Pharmingen, San Diego, CA, USA), anti-CCR4 PerCP-Cy5.5 (BD Pharmingen, San Diego, CA, USA), anti-CXCR3 BV711 (BD Pharmingen, San Diego, CA, USA), and anti-CCR2 PE (Biolegend, San Diego, CA, USA). Panel C: Anti-CD3 BV650 (BD Pharmingen, San Diego, CA, USA) and anti-CD8 FITC (BD Pharmingen, San Diego, CA, USA); anti-CD4 APC-Cy7 (BD Pharmingen, San Diego, CA, USA), anti-CD25 PECF584 (BD Pharmingen, San Diego, CA, USA), anti-TNFR2 Biotin-Streptavidin AF700 (BD Pharmingen, San Diego, CA, USA), anti-IL-6R PE-Cy7 (Biolegend, San Diego, CA, USA), anti-CD38 PerCP-Cy5.5 (BD Pharmingen, San Diego, CA, USA), anti-CD69 PE (Biolegend, San Diego, CA, USA), and anti-GARP BV 711 (BD Pharmingen, San Diego, CA, USA). Cells were stained in diluted antibodies at appropriate concentration in dPBS with 5% human serum (staining buffer). Cells were stained at room temperature for 15 min at a final concentration of 10^7^ cells per 150 μL of antibody cocktail. Cells were then washed twice with staining buffer.

After primary staining, a fixable dead cell dye (Life Technologies, USA) was used to distinguish between dead and live cells. Following the fixation and permeabilisation of cells using a fixation/permeabilisation buffer kit (eBioscience, San Diego, CA, USA), intracellular staining with the appropriate antibodies was performed at a final concentration of 10^7^ cells per 150 μL and incubated for 15 min at room temperature: Panel A—anti-FOXP3 APC (eBioscience, San Diego, CA, USA) and anti-STAT3 Phospho (Tyr705) BV421 (Biolegend, San Diego, CA, USA); Panel B—anti-FOXP3 APC (eBioscience, San Diego, CA, USA); Panel C—anti-FOXP3 APC (eBioscience, San Diego, CA, USA), anti-Ki67 BV786 (BD Pharmingen, San Diego, CA, USA). Cells were then washed twice and re-suspended in dPBS with 1% paraformaldehyde (PFA, Sigma-Aldrich, St. Louis, MI, USA). Flow cytometry data were acquired on a Becton Dickinson LSR II flow cytometer using FACS Diva software, acquiring a minimum of 100,000 events per sample. Fluorescence minus one (FMO) controls and isotype-matched immunoglobulins were used to enable accurate gating. Data were analysed using Flowjo software (TreeStar, Woodburn, OR, USA). The proportion of Treg cells was determined as the percentages of CD25^hi^FOXP3^+^ cells among CD4^+^ lymphocytes and T effectors (Teffs) as CD25^-^FOXP3^-^ T cells among CD4^+^ lymphocytes.

### 2.9. Statistical Analysis of Data

#### 2.9.1. Volcano Plot

Unpaired *t*-tests were conducted with Bonferroni testing correction between two groups on each of the log_2_-transformed immune factors: the proportion of 16 phenotypic markers on Tregs and Teffs, the concentration of 28 * serum soluble factors, demographic characteristics, clinical biomarker levels and risk indices scores. For each immune factor, two pairwise contrasts of means—malignant tumour versus normal ovaries, benign tumour versus normal ovaries and malignant tumour versus benign tumour—and their standard errors of difference were calculated, and the results of the pairwise t-tests were summarised in a volcano plot of statistical significance, –log_10_(*p*-value) versus fold change, namely, the difference in the group means of the log_2_-transformed immune factor values. Immune factors with contrasts greater than a 2-fold change or with a *p*-value < 0.05 are labelled. * Interleukin-17 was not statistically analysed as all values were below the detection limit and it was therefore excluded from analysis.

#### 2.9.2. Principal Component Analysis (PCA)

PCA was performed to describe the variance of cytokine concentrations and proportions of phenotypic markers. All variables were transformed using log_2_ to reduce skewness prior to PCA. The resulting PCA component scores were extracted for analysis of the association with the study patients categorised according to tumour status and different responses to chemotherapy. For a robust mathematical model with a reliable predictive accuracy, the values of the immune markers to be evaluated for diagnostic accuracy should ideally be above 0.5 or close to 1.0 [54].

#### 2.9.3. Receiver Operator Characteristic (ROC)

ROC analyses were performed to determine the predictive value of each biomarker individually or in combination. The area under curve (AUC) was obtained for each immune marker, and predictive probability for immune markers in combination were also determined using binomial logistic regression. Good risk prediction models will have an AUC greater than 0.7 [55], and the most informative biomarker will increase the AUC by 0.005 or more [56]. Statistical significance was defined as *p* < 0.05 or <0.0001 where appropriate, and 95% confidence interval (CI) is also reported.

Continuous variables were evaluated by the Student *t*-test or an ANOVA test. Categorical variables were evaluated by the *χ^2^* test or Fisher exact test. We performed univariate followed by multivariate logistic regression analyses with odds ratios (ORs) or adjusted ORs, and 95% confidence intervals (CIs) for evaluating immune factors in predicting adnexal masses.

Graphpad Prism version 9.0 and SPSS (IBM 25.0) were used for all analyses.

## 3. Results

Table 2 summarises the baseline characteristics of all study patients. All patients with ovarian cancer had Stage III-IV high-grade serous adenocarcinoma (HGSOC) (*n* = 33) and the majority of patients with a benign ovarian mass (*n* = 12) had mainly serous cystadenomas (75%), while patients undergoing risk reduction surgery for a genetic mutation or family history of hereditary breast or ovarian cancer (*n* = 21) had pathologically confirmed normal ovaries. The mean ages of patients with ovarian cancer and those with benign ovarian mass were similar, while patients with normal ovaries were relatively younger (Table 2). There was no significant difference in all other blood component counts, except that the mean of total lymphocytes was significantly lower in ovarian cancer patients (Table 2).

**Figure 2:**
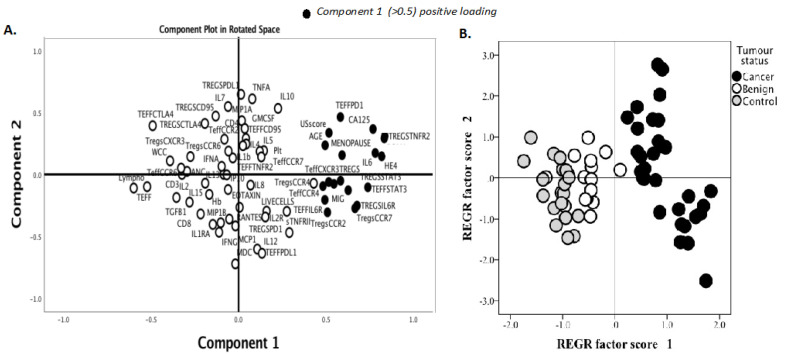
Principal component analysis (PCA) of serum soluble factor levels and T cell phenotypic markers to identify immune factors that are predictive of malignancy. Pre-treatment blood was withdrawn from 66 patients: 33 patients with ovarian cancer, 12 benign ovarian mass and 21 with normal ovaries. A total of 28 serum soluble factors were measured using multiplex bead immunoassay and 16 phenotypic markers (surface and intracellular) on regulatory T cells (Tregs) and effector T cells (Teff) were identified and quantified by flow cytometry. Demographic data, serum CA125, HE4 and blood components were included in the analysis. (**A**) Data reduction was conducted using principle component analysis. The clusters of immune markers with reliable predictive accuracy (values of diagnostic parameters above 0.5) were labelled as components 1 (black dots). (**B**) Component scores were applied to individuals in the study according to their disease categories.

**Figure 3:**
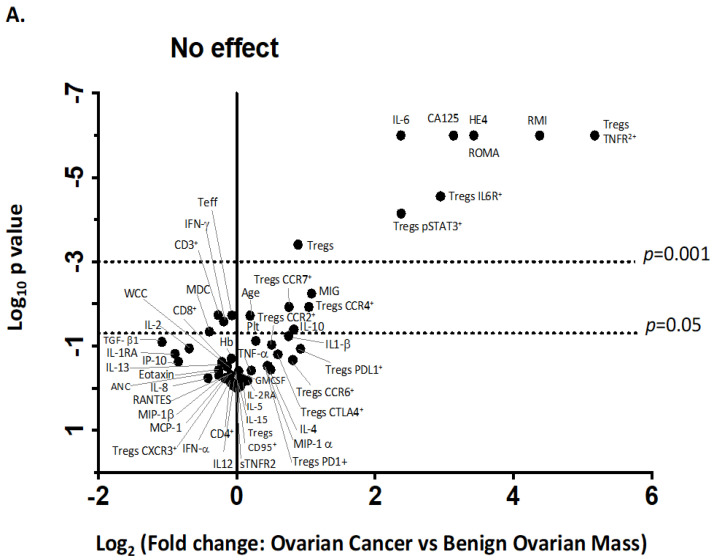

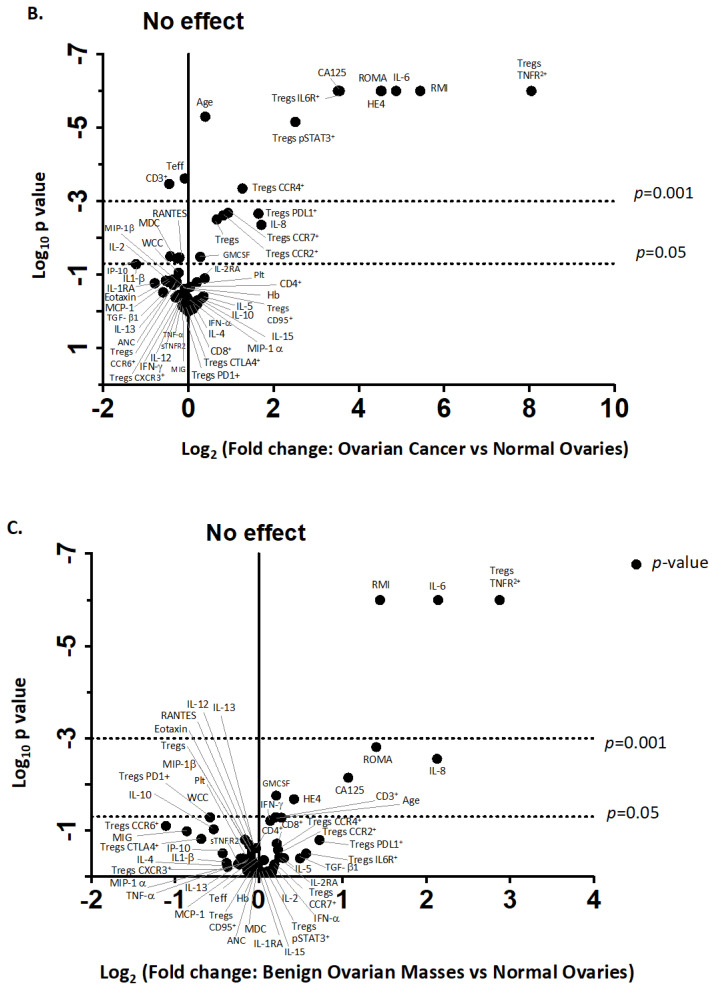
Volcano plot for the identification of discriminating biomarkers. Pre-treatment blood was withdrawn from 33 patients with ovarian cancer, 12 with benign ovarian mass and 21 with normal ovaries. A total of 28 serum-soluble factors measured using multiplex bead immunoassay and 16 phenotypic markers on Tregs and Teff quantified by flow cytometry as well as demographic data, blood components, serum CA125 and HE4 were included in analysis. Each circle corresponds to one study factor. The figure represents the negative log_10_ *p*-values plotted against the log_2_-fold change of study factors, namely, the difference between two groups: (**A**) cancer versus benign ovarian mass, (**B**) cancer versus normal ovaries and (**C**) benign versus normal ovaries. Study factors with contrasts greater than a 2-fold change or with a *p*-value < 0.05 are labelled.

**Figure 4:**
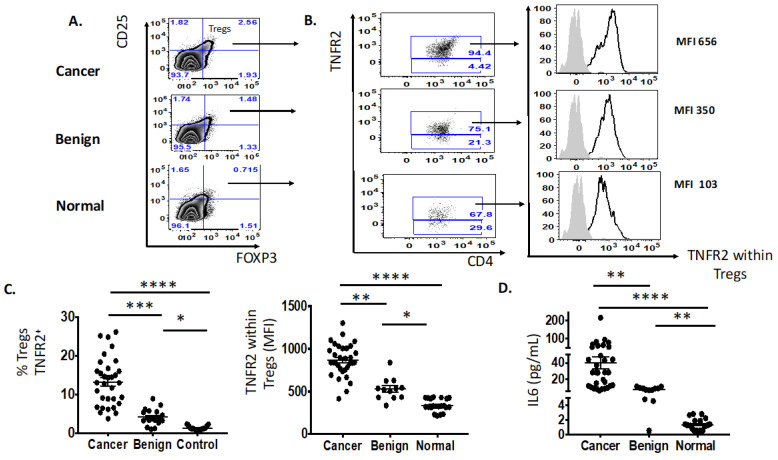
Increased proportion of Tregs TNFR2^+^ and IL-6 in the pre-treatment peripheral blood of patients with advanced HGSOC (*n* = 33) compared to benign (*n* = 12) and normal ovaries (*n* = 21). (**A**) Flow cytometry plot of proportion of Tregs, CD25^hi^FOXP3^+^. (**B**) Flow cytometry plot of frequency (%) and expression (MFI, median fluorescence intensity) of TNFR2^+^ Tregs in advanced HGSOC patients (*n* = 33) compared to those with benign ovarian masses (*n* = 12) and normal ovaries. (**C**) The proportion of Tregs TNFR2^+^ (in colored font and top right-hand column with black arrow) and level of expression (MFI) of TNFR2-expressing Tregs between three study groups. (**D**) The level of IL-6 (pg/mL) in the serum between three study groups. One-way ANOVA and Dunn’s multiple comparison test (post hoc); * *p* < 0.05, ** *p* = 0.001–0.01, *** *p* = 0.0001–0.001, **** *p* < 0.0001 (error bars—SEM).

We identified two immune factors, namely, TNFR2^+^ Tregs and IL-6, as highly correlated new variables, in addition to the conventional biomarkers and tests known to be discriminative between the study groups (Figure 2). We then analysed all these study factors using volcano plot analysis (Figure 3), which summarises the significant factors with greater than 2-fold change in a pair-wise comparison between the three groups: malignant versus benign ovarian masses, malignant versus normal ovaries, and benign versus normal ovaries. Immune factors TNFR2^+^ Tregs and IL-6, CA125 and HE4 were consistently seen as significant discriminatory factors able to distinguish across all three pair-wise combination groups (Figure 3). These two immune factors were then individually assessed for their ability to distinguish between the three groups using one-way ANOVA, followed by Dunn’s post-hoc test (Figure 4). The median proportion of TNFR2^+^ Tregs in the peripheral blood of patients with ovarian cancer was 8-fold and 34-fold higher compared to benign ovarian masses and normal ovaries, respectively (Figure 4). The median level of expression of TNFR2, as measured by median fluorescence intensity (MFI) on Tregs (CD25^hi^FOXP3^+^), was also increased in ovarian cancer patients compared to benign ovarian masses and normal ovaries, respectively (840.6 versus 526.4 versus 326.4). The concentration of IL-6 was higher in the serum of patients with ovarian cancer compared to benign ovarian masses or normal ovaries (median IL-6: 28.3 vs. 6.4 vs. 1.2 pg/mL, *p* < 0.0001) (Figure 4). All patients with advanced HGSOC had a detectable concentration of IL-6 in their serum (range: 5.6–215.88 pg/mL), while up to one-third of patients with normal ovaries had concentrations of IL-6 below the detection range (less than 0.5 pg/mL).

**Figure 5:**
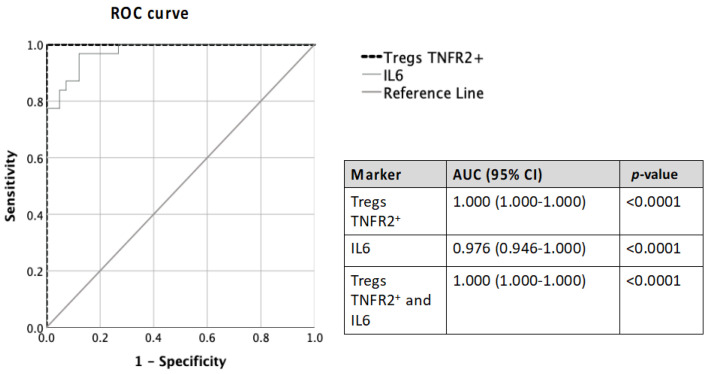
Comparison of ROC-AUC values of pre-treatment Tregs TNFR2^+^ and IL-6 alone and in combination in predicting ovarian malignancy.

ROC (receiver operator characteristic) analysis of TNFR2^+^ Tregs and IL-6 was performed to evaluate their predictive ability to distinguish ovarian malignancy compared to current clinical tests (RMI and ROMA) and biomarkers (CA125 and HE4) (Table 3 and Table 4). The use of data on the pre-treatment proportion of TNFR2^+^ Tregs and IL-6 concentration showed excellent to good predictive ability in distinguishing between malignant and non-malignant patients (AUC—1.000 and 0.976, respectively) (Figure 5). Moreover, the combination of IL-6 and TNFR2^+^ Tregs achieved an excellent predictive ability (AUC 1.000) (Figure 5) in identifying patients with malignant tumors, and this was superior to AUC values for CA125 and HE4 (AUC value ranges 0.986–0.997) (Table 3 and Table 4). 

We observed a higher proportion of CCR4^+^ and CCR7^+^ Tregs, migratory-related chemokines in the pre-treatment blood of patients with ovarian cancer compared to benign ovarian masses and normal ovaries (Figure 2 and Figure 3); however, these markers were not able to discriminate between those with benign tumors and normal ovaries. ROC analyses of these markers only achieved modest predictive ability. Consistent with the elevated proportion of migratory-related chemokine receptors, we also observed higher concentrations of chemokine ligand CCL22/MDC (macrophage-derived chemokine); however, we did not achieve a satisfactory predictive value for ovarian malignancy (Table 3). Similarly, we also found the upregulation of PD-L1 and PD-1 on Tregs and Teffs, respectively, in the pre-treatment blood of ovarian cancer patients, but these were not discriminatory between the three study groups (Figure 3). We also observed in our study that STAT3-expressing Tregs consistently appeared in a cluster with TNFR2-expressing Tregs in PCA and volcano plot analysis for the discrimination of ovarian malignancy (Figure 2 and Figure 3). The proportion of STAT3^+^ Tregs only achieved a modest predictive value for discrimination of ovarian malignancy. Consistent with the hypothesis that the ovarian cancer microenvironment is influenced by inflammatory and immunosuppression molecules, we found higher concentrations of TGF-β, IL10 and IL8, but these soluble factors did not achieve satisfactory predictive values to discriminate ovarian malignancy (Table 3).

Considering that IL-6 signalling is mainly mediated by the signal transducer and activator of transcription-3 (STAT3) pathway, we further explored whether an altered phosphorylated state of the tyrosine residue 705 (pY705) STAT3 protein may also influence the expression of TNFR2 phenotype in the ovarian cancer microenvironment. We found a positive correlation between the log proportion of TNFR2 and the log proportion of phosphorylated STAT3 (pSTAT3) in freshly stained PBMCs from all 33 patients with advanced HGSOC (Figure 5).

**Figure 6:**
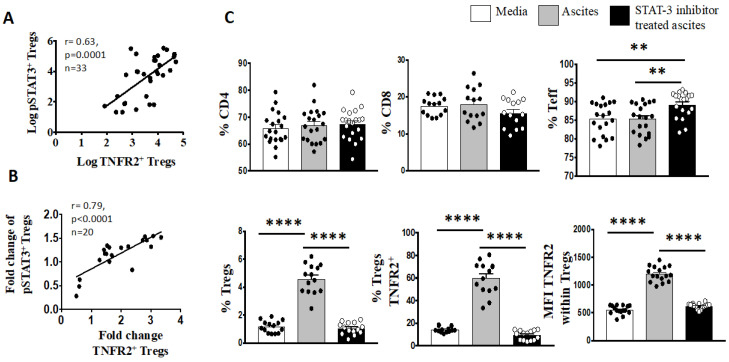
Blockade of pY705-STAT3 signaling decreases percentage of Tregs, as well as TNFR2+ Tregs and TNFR2 expression. (**A**) Peripheral blood mononuclear cells (PBMCs) from patients with advanced HGSOC (*n* = 33) were isolated by Ficoll density centrifugation and freshly stained for surface and intracellular markers CD3, CD4, CD25, TNFR2, FoxP3 and pSTAT3 and analysed with flow cytometry. (**B**,**C**) PBMCs from healthy donors (*n* = 20) were isolated by Ficoll density centrifugation and incubated in vitro in media or cell-free ascites from advanced EOC patients for 48 h. pY705-STAT3 activity was inhibited by the addition of 50 μM S3I-201 into the culture at day 0. In untreated media and ascites, vehicle control DMSO at 0.05% was added at day 0. Cells were washed, labeled for surface and intracellular markers CD3, CD4, CD25, TNFR2, FoxP3 and pSTAT3, and analysed with flow cytometry. (**A**,**B**) Correlation of the percentage of pSTAT3+ Tregs to TNFR2+ Tregs (log) in patients with advanced HGSOC. B. Correlation of the fold change of pSTAT3+ Tregs to TNFR2+ Tregs in healthy donors PBMC following incubation in ascites of EOC patient, *p* < 0.05 is significant. (**C**) The frequency (%) of CD4, CD8, Teff and Tregs as well as the percentage of TNFR2 and level of TNFR2 expression (median fluorescence intensity, MFI) within Tregs within media (white bar), ascites (grey bar) and following blockade with STAT3 inhibitor (black bar). ** *p* = 0.001–0.01, **** *p* < 0.0001—one-way ANOVA and Dunn’s multiple comparison test (post-hoc). Data are pooled from two independent experiments (error bars—SEM).

We have shown in a recently published paper that IL-6 in ovarian cancer ascites promotes the upregulation of TNFR2 on T cells, predominantly on Tregs, with the neutralisation of the bioactive IL-6 decreasing the proportion of TNFR2 [57]. We next explored whether the proportion of the pSTAT3 was also correlated with TNFR2-expressing Tregs in the presence of high levels of IL-6. We incubated PBMCS from 20 healthy donors in cell-free ascites obtained from advanced HGSOC patients (with a known concentration of IL-6) for 48 h using a similar in vitro culture system as published in Kampan et al. [57]. Following ascites incubation, the fold-change in TNFR2-expressing Tregs that was observed in the previous study [57] correlated positively with the fold-change of pSTAT3-expressing Tregs (Figure 6). We investigated whether the regulation of STAT3 may account for the modulation of TNFR2 expression on Tregs by IL-6. Using a similar in vitro culture system, we analysed the phosphorylation state of the STAT3 protein on T cells upon the conditioning of PBMCs from healthy donors in S3I-201-treated and untreated malignant cell-free ascites for 48 h. Following analyses by flow cytometry, the overall fraction of CD4^+^ and CD8^+^ T cells following conditioning in ascites and blockade in the presence or absence of a STAT3 inhibitor (S3I-201) showed no significant difference compared to control media (Figure 6). By contrast, within CD4^+^ T cells, there was a clear increase in Teffs and decrease in Tregs following S3I-201-treated ascites compared to untreated ascites (Figure 5). Following pSTAT3 signalling inhibition in ascites, the proportion of Tregs as well as the proportion of TNFR2^+^ Tregs and the level of TNFR2 expression within Tregs decreased compared to untreated ascites.

## 4. Discussion

The present study shows for the first time that the combination of suppressive and pro-inflammatory immune markers, TNFR2-expressing Tregs and IL-6, found to be over-expressed in the peripheral blood of advanced high-grade serous ovarian cancer patients at diagnosis, are the most informative immune markers able to discriminate between those with ovarian cancers, benign ovarian masses and normal ovaries, and have superior predictive abilities compared to conventional tests and current biomarkers when used in combination. We found a positive correlation between the proportion of TNFR2-expressing Tregs and STAT3-expressing Tregs in all patients with advanced HGSOC. We further investigated their relationship in an in vitro culture system by incubating PBMCs from healthy donors in malignant ascites containing high levels of IL-6. Confirming previous results, IL-6 in malignant ascites promoted TNFR2 expression on Tregs [57], and herein we further identified that the IL-6-mediated TNFR2 modulation of Tregs may operate via the constitutive phosphorylation of the STAT3 Y705.

In our study, we assessed many soluble factors and phenotypic markers on T cells in the pre-treatment blood to identify discriminatory biomarkers using two robust statistical methods. We found that the concentration of IL-6 in the serum at baseline in malignancy was increased by 4- and 23-fold compared to those with benign ovarian masses and normal ovaries, respectively. Similarly, we found that the proportion of TNFR2^+^ Tregs was elevated in the peripheral blood of patients with advanced-stage HGSOC at diagnosis by 8- and 34-fold when compared to patients with benign ovarian masses and normal ovaries, respectively. IL-6 is a multifunctional cytokine that has long been linked with malignant transformation, cancer cell proliferation and progression of HGSOC [30,58]. The sources of IL-6 are from both malignant and normal cells [59], with PBMC from ovarian cancer patients reported to secrete higher concentrations of IL-6 compared to healthy controls, and IL-6 is also expressed at different concentrations within ovarian cancers of various grades and stages [60,61,62,63]. Similarly, TNFR2 expressed on normal and malignant cells has been deemed both as a product of oncogene and an inducer of Treg expansion [64,65]. Therefore, it is not surprising that a high expression of TNFR2 has been reported in various tumour types, including ovarian cancer. The few studies to have looked at TNFR2 expression on immune cells in human peripheral blood have also suggested negative clinical associations. Thus, while TNFR2-expressing Tregs have been found in abundance in the peripheral blood of patients with leukaemia [66] compared to healthy controls, the higher expression of TNFR2 in the blood of patients with lung cancer correlated with advanced stages and invasiveness of the disease [67].

To the best of our knowledge, this is the first study to show that IL-6 and TNFR2^+^ Tregs in combination may be used as a diagnostic tool to indicate patients with ovarian cancers. The discriminatory ability of these immune biomarkers was superior to existing clinical risk indices and biomarkers. Moreover, the combination of the proportion of TNFR2^+^ Tregs and the concentration of IL-6 achieved excellent predictive ability (AUC value 1.000) to predict ovarian malignancy, which is superior to existing conventional biomarkers. The discriminative ability of IL-6 for ovarian cancer in the pre-treatment serum of our patients has been validated in an independent cohort of advanced HGSOC [28]. Further validation studies are needed to confirm the combined predictive ability of these immune biomarkers for discriminating ovarian cancer. The use of flow cytometry has long been the mainstay of the diagnostic regimen for blood-related cancers [68]. Overall, the best practice to achieve a comprehensive diagnosis should include clinical examination, morphologic assessment, flow cytometry analysis, and other relevant tests, such as cytological evaluation and genetic testing [68,69].

These new biomarkers may provide additional choices in the selection of an optimal panel of biomarkers to be tested in future screening for ovarian cancer, hence improving the lack of sensitivity, specificity, and accuracy as a result of the current reliance on one or a couple of biomarkers. The advantage of a blood biomarker is its inherent objective nature, which facilitates reproducibility, as compared to the subjective assessment of operator-dependent morphologic features of ovarian neoplasms. The patients in our study constituted a homogenous group of patients at first diagnosis with advanced stages of ovarian cancer (FIGO Stage III/IV), and therefore represent only one, although the commonest, subtype of ovarian cancer presentation. Therefore, the conclusions of this study may not be applicable to patients in early stages of ovarian cancers or other subtypes, and this requires further evaluation. As IL-6 plays a critical role in promoting cancer cell growth and survival [30,70,71], the abundance of these biomarkers is observed not only in primary epithelial ovarian cancers, but also in a variety of other solid tumours including breast, lung and colorectal cancers, which can make diagnosis particularly challenging [30,64,70,72,73,74,75]. The possibility of metastatic breast, lung, or bowel cancers merits consideration in the establishment of differential diagnoses for a woman with suspicious ovarian mass and elevated IL-6.

The IL-6- and TNFR2-associated pathways are emerging as critical mediators of inflammation-associated cancers, including ovarian malignancy [30,64,70,71,76,77,78,79,80]. In our study, we found a significant overlapping contribution of TNFR2-expressing Tregs and IL-6 in predicting ovarian malignancy. This may be explained by the fact that HGSOC is a heterogeneous cancer [22], with constitutive activation of multiple signalling pathways including STAT3 and NFκB, which may explain its aggressive nature and early invasiveness [33,81]. We found in our previous study that TNFR2 expression can be promoted on T cells incubated in cell-free ascites, and this is most likely mediated by IL-6, as the neutralisation of IL-6 decreased TNFR2 expression, mainly on Tregs [57].

Hamilton et al. looking at the molecular mechanism of the induction of TNFR2 in colon cancer cell lines, reported that TNFR2 induction, although it requires TNF in combination with IL-6 to enhance expression of TNFR2, may be induced sufficiently by IL-6-mediated STAT3 activation alone [82]. The STAT3-mediated increase in TNFR2 expression on colon cancer cell lines was reduced by SOCS3, a cytokine-inducible STAT3 inhibitor [82]. In this study, as observed in our in vitro culture experiments, we found that the increased proportion of TNFR2 on Tregs may be mediated by IL-6 via the STAT3 signalling pathway. Therefore, both IL-6 and TNFR2 may contribute to the tumour-promoting roles of STAT3, hence targeting the IL-6-signaling pathway may help to decrease maximally suppressive TNFR2^+^ Tregs. In addition, TNFR2-targeted inhibitors may eliminate not only cancer cells, but also Tregs, which will contribute to immunosuppression and may help to restore the suppressed tumour killing ability of effector T cells [64,70,71,83,84]. 

## 5. Conclusions

Pre-treatment serum IL-6 and peripheral blood TNFR2^+^ Tregs may be potential clinical biomarkers usable to discriminate patients with malignant compared to benign ovarian cancer masses, and the relationship between IL-6 and TNFR2^+^ Tregs is likely to be mediated via the STAT3 signalling pathway.

## Figures and Tables

**Table 1:** Study inclusion and exclusion criteria.

Inclusion Criteria	Exclusion Criteria
Age 18–80	Age < 18 or >80 years
Signed written informed consent	Unable to give informed consent
Newly diagnosed, Stage III–IV, high-grade serous ovarian cancer (HGSOC) or benign ovarian tumour or normal ovaries, which are pathologically confirmed	Pregnant Cancer other than Stage III–IV HGSOC Concurrent other active cancers Concurrent significant pre-existing major medical conditions (such as heart, liver or vascular diseases)
No prior chemotherapy or radiotherapy	Major surgery, open biopsy or significant trauma or injury within 28 days prior to sampling
	Receiving NSAIDS, anti-inflammatory steroids or immunosuppressive agents within 14 days prior to sampling
	Active inflammation, significant trauma or open wound

**Table 2:** Characteristics of study patients according to tumour status.

	Ovarian Cancer *n* = 33	Benign Ovaries *n* = 15	Normal Ovaries *n* = 21	*p*-Value
Demographic data				
Mean age ± SD (years) Median Age range	60.1 ± 1.59 60 41–83	54.8 ± 3.07 55.5 38–73	51 ± 2.20 48 40–84	^a^*p* = 0.30 ^b^ *p* = 0.001 ^c^ *p* = 0.66
Mean BMI ± SD (kg/m^2^) Median BMI range	29.69 ± 0.85 28.5 24–40	29.73 ± 1.35 28 24–40	30.05 ± 1.20 28 24–43	ns
Cancer characteristics				
Histology	High-grade papillary serous adenocarcinoma	Serous cystadenoma- *n* = 12 Fibrothecoma-*n* = 3	No pathology	
Stage	IIIA-1 IIIB-2 IIIC-28 IV-2	NA	NA	
Blood component counts				
Mean Hb (g/L) Median	121.4 ± 16.8 122	121.2 ± 20.8 121	123 ± 15.7 122	ns
Mean platelet Median	327.8 ± 24.3 296	261 ± 21.1 229	267 ± 12.8 263	ns
Mean WCC (×10^9^/L) Median	8.51 ± 2.89 8.5	9.30 ± 3.23 10.2	10.16 ± 3.3 10.2	ns
Mean absolute neutrophil count (×10^9^/L) Median	5.92 ± 3.09 5.04	5.93 ± 2.89 5.49	6.86 ± 3.35 6.28	ns
Mean total lymphocytes (×10^9^/L) Median	1.88 ± 0.58 1.8	2.72 ± 0.88 2.58	2.43 ± 0.65 2.26	^a^*p* = 0.005 ^b^ *p* = 0.002 ^c^ *p* = 0.99

±SD-Standard deviation, ^a^ cancer vs. benign *t*-test, ^b^ cancer vs. normal *t*-test, ^c^ benign vs. normal *t*-test, ns = *p*-value ≥ 0.05 = statistically non-significant.

**Table 3:** Comparison of ROC-AUC values of demographic and pre-treatment immune factors for patients with malignant and non-malignant ovarian masses.

Factors	AUC ^a^	SE	*p*-Value ^b^	95% CI
**Demographic data**				
Age	0.760	0.057	0.001	0.649–0.872
Menopause	0.801	0.054	0.001	0.695–0.908
**Laboratory parameters**				
Haemoglobin (Hb)	0.422	0.069	ns	0.287–0.557
Platelet	0.635	0.070	ns	0.498–0.771
White cell count (WCC)	0.366	0.067	ns	0.236–0.497
Absolute neutrophil Counts (ANC)	0.399	0.069	ns	0.263–0.535
Total lymphocytes	0.183	0.049	0.001	0.088–0.279
**Clinical tests and biomarkers**				
CA125	0.986	0.010	<0.0001	0.966–1.000
HE4	0.997	0.001	<0.0001	0.988–1.000
**Tregs phenotypes**				
CD95^+^	0.630	0.068	ns	0.497–0.763
CTLA-4^+^	0.608	0.072	ns	0.467–0.749
IL6R^+^	0.855	0.045	0.001	0.767–0.944
PD-L1^+^	0.710	0.061	0.002	0.590–0.831
PD-1^+^	0.613	0.073	ns	0.470–0.755
STAT3^+^	0.822	0.048	0.001	0.729–0.915
TNFR2^+^	1.000	0.001	<0.0001	1.000–1.000
CCR4^+^	0.714	0.062	0.002	0.593–0.836
CCR6^+^	0.524	0.07	ns	0.386–0.662
CXCR3^+^	0.505	0.073	ns	0.362–0.648
CCR7^+^	0.701	0.064	0.004	0.576–0.826
CCR2^+^	0.707	0.064	0.003	0.582–0.833
**Teff phenotypes**				
CD95^+^	0.600	0.069	ns	0.464–0.736
CTLA-4^+^	0.476	0.08	ns	0.320–0.633
IL6R^+^	0.696	0.063	0.005	0.572–0.820
PDL1^+^	0.46	0.071	ns	0.320–0.600
PD-1^+^	0.846	0.046	0.001	0.756–0.935
STAT3^+^	0.864	0.042	0.001	0.783–0.946
TNFR2^+^	0.462	0.079	ns	0.307–0.617
CCR4^+^	0.646	0.074	ns	0.502–0.791
CCR6^+^	0.352	0.074	ns	0.207–0.496
CXCR3^+^	0.590	0.076	ns	0.440–0.739
CCR7^+^	0.495	0.080	ns	0.339–0.652
CCR2^+^	0.475	0.078	ns	0.303–0.611

AUC—area under curve, SE—standard error, CI—confidence interval. ^a^ Null hypothesis: true area = 0.5, ^b^ *p*-value ≥ 0.05 = statistically non-significant (ns).

**Table 4:** Comparison of ROC-AUC values of pre-treatment soluble factor concentration for patients with malignant and non-malignant ovarian masses.

Soluble Factors	AUC ^a^	SE	*p*-Value ^b^	95% CI
IL-1b	0.611	0.067	ns	0.479–0.743
IL-10	0.536	0.070	ns	0.398–0.674
IL-13	0.42	0.070	ns	0.282–0.558
IL-6	0.976	0.014	<0.0001	0.948–1.000
IL-12	0.415	0.071	ns	0.275–0.555
RANTES	0.377	0.067	ns	0.246–0.507
EOTAXIN	0.438	0.070	ns	0.300–0.576
MIP-1α	0.536	0.071	ns	0.397–0.675
GMCSF	0.58	0.072	ns	0.44–0.721
MIP-1β	0.421	0.070	ns	0.284–0.559
MCP-1	0.471	0.074	ns	0.326–0.617
IL-15	0.517	0.069	ns	0.381–0.652
IL-5	0.565	0.070	ns	0.428–0.702
IFN-γ	0.367	0.068	ns	0.234–0.501
IFN-α	0.526	0.069	ns	0.391–0.66
IL-1RA	0.42	0.070	ns	0.283–0.556
TNF-α	0.538	0.068	ns	0.405–0.672
IL-2	0.431	0.067	ns	0.299–0.563
IL-7	0.526	0.069	ns	0.39–0.661
IP-10	0.427	0.077	ns	0.276–0.579
IL-2RA	0.585	0.069	ns	0.449–0.721
MIG	0.602	0.067	ns	0.472–0.733
IL-4	0.533	0.069	ns	0.398–0.668
IL-8	0.743	0.025	0.001	0.618–0.868
sTNFR2	0.528	0.07	ns	0.391–0.665
MDC	0.36	0.069	0.042	0.224–0.495
TGF-β1	0.355	0.067	0.036	0.224–0.486

AUC—area under curve, SE—standard error, CI—confidence interval. ^a^ Null hypothesis: true area = 0.5, ^b^ *p*-value ≥ 0.05 = statistically non-significant (ns).

## Data Availability

The data presented in this study are available on request from the corresponding author.
